# Characteristics and hospitalizations among children on public assistance in Japan: A population‐based cohort study

**DOI:** 10.1111/ped.70005

**Published:** 2025-06-10

**Authors:** Daisuke Nishioka, Keiko Ueno, Shiho Kino, Naoki Kondo

**Affiliations:** ^1^ Department of Social Impact Assessment, Graduate School of Medicine and School of Public Health Kyoto University Kyoto Japan; ^2^ Department of Medical Statistics, Medical Research and Development Center Osaka Medical and Pharmaceutical University Osaka Japan; ^3^ Department of Social Epidemiology, Graduate School of Medicine and School of Public Health Kyoto University Kyoto Japan; ^4^ Department of Preventive Oral Health Care Sciences, Graduate School of Medical and Dental Sciences Institute of Science Tokyo Tokyo Japan

**Keywords:** hospitalization, Japan, poverty, public assistance, public health

## Abstract

**Background:**

Child poverty is a critical issue in Japan. While public assistance (*seikatsu‐hogo*) alleviates financial barriers to healthcare, the multidimensional nature of poverty, including social and environmental factors, may still affect children's health. Although previous research has explored health disparities among adults receiving public assistance, little is known about the current conditions and healthcare needs of child recipients. This study aimed to describe the characteristics of children on public assistance and identify the incidence and predictors of hospitalization among them.

**Methods:**

This retrospective cohort study included children receiving public assistance in six Japanese municipalities. We extracted data on children's age, sex, household composition, siblings, guardians' employment status, timing of public assistance receipt, nationality, and disability certificates as of April 2016 and observed them for 1 year. The primary outcome was all‐cause hospitalization. Poisson regression analyses were performed to identify predictors of hospitalization.

**Results:**

We analyzed data from 1990 children receiving public assistance. Overall, 4.6% were hospitalized during the observation period, with higher rates in younger children and those with disabilities. Hospitalization incidence was varied across households conditions and residing municipalities.

**Conclusions:**

Children on public assistance in Japan may face higher hospitalization risks, and the differences were observed across household conditions and residential areas. Further research using more detailed data should explore child poverty and health, including both recipients and non‐recipients of public assistance.

## INTRODUCTION

The eradication of poverty is an important aspect of the Sustainable Development Goals (SDGs) adopted by United Nations member states in 2015.^1^ The influence of poverty on children's development and health has been the focus for many decades, and evidence for the societal and public health implications of poverty has been established.^2^, ^3^, ^4^ As poverty is a strong determinant of health, governments in many countries provide welfare support with financial aid for impoverished people, and the programs include full or partial exemptions from healthcare costs.^5^ Recently, the dynamic and multidimensional nature of poverty has been increasingly recognized.^6^, ^7^ This concept encompasses not only financial difficulties but also challenges in other fundamental aspects of well‐being, such as health, education, and social relationships. These nonfinancial dimensions of poverty, often referred to as the social determinants of health, play a critical role in shaping children's health outcomes.^8^, ^9^

In recent decades, the Japanese government has been grappling with child poverty. Japan's child relative poverty rate was reported to be 14.0% in 2020, which is higher than the average among the member nations of the Organization for Economic Co‐operation and Development (OECD).^10^ In particular, approximately half of the children living with single parents live below the poverty line.^11^ One of the measures that can potentially address child poverty is the Child Medical Expense Subsidy (*kodomo‐iryohi‐josei*), which can exempt out‐of‐pocket medical expenses of children.^12^ However, given the potential influence of multidimensional poverty, financial welfare programs for healthcare alone may not fully compensate for socioeconomic health risks. Kato et al. reported that the contribution of Child Medical Expense Subsidy to children's health is limited.^13^ Further, findings of Abe 2013 showed that the hospitalization rate of poor children cannot be sufficiently mitigated even if the programs of Child Medical Expense Subsidy were well‐established.^14^

In a global perspective, as a measure to help people in poverty other than the medical expense subsidy, although several governmental welfare programs were established, their impact on children's health has been controversial. For example, in the United States, welfare reforms, such as the Temporary Assistance for Needy Families (TANF) program, can improve the children's reported health outcomes,^15^ but the longer they receive the assistance, the potential higher risks for adverse health outcomes, including increased rates of chronic illness and hospitalization.^16^

In Japan, a comparable governmental welfare program, known as public assistance or *seikatsu‐hogo*, benefits households living below the poverty line without any assets. The municipal welfare office conducts rigorous tests and determines whether people will receive benefits,^17^ and approximately 1.6% of the entire population in Japan received public assistance in 2021 while the child poverty rate was reported to be 14% of the entire population. Using public assistance can receive the benefits, including full exemption from the payment for healthcare utilization, households' minimum income benefits, and benefits for the children's education.^17^ Previous research has shown that the recipients of public assistance have disadvantageous health statuses;^18^ however, evidence of the health statuses of children receiving public assistance using population‐based data in Japan is still sparse.^19^, ^20^ Only a study using population‐based data reported that children receiving public assistance are at higher risk for school refusal.^19^ To investigate if the public assistance program has a protective effect on children's health, we need to compare the health statuses of children across the groups of receiving public assistance or not; healthcare data, including both populations in Japan, is not available at present. Therefore, health statuses of each population need to be clarified separately and compared using currently available data.

A previous research has revealed that children receiving public assistance have a higher prevalence of chronic diseases, especially those living in single‐parent households.^20^ However, evidence determined using medical claims data for outpatient care does not always reflect the severity of children's health, and children with mild symptoms without consultation could not be included in the outpatient claim data, leading to over/underestimation of children's medical needs. Therefore, it still remained unclear whether this evidence reflects a genuinely higher prevalence of disease or more frequent healthcare utilization in single‐parent households or if children from nonsingle‐parent households are underserved from medical care. Hospitalization data, unlike outpatient claims, provides a more reliable measure of healthcare needs, as it reduces the bias that may arise from varying healthcare‐seeking behaviors for less severe conditions.^21^, ^22^ By focusing on hospitalization, this study aims to provide a clearer picture of the healthcare needs of children receiving public assistance who were not well described and understood by society as a whole.

Thus, the aim of this study is twofold: first, to describe the demographic characteristics of children receiving public assistance in Japan; and second, to identify the incidence and determinants of hospitalization in this population, using municipal public assistance data.

## METHODS

### Design and study participants

This retrospective cohort study included all children under 15 years of age who had received public assistance in six Japanese municipalities as of April 2016. The characteristics of the six municipalities were shown in Table S1.

### Data sources

For baseline data, we used a database of public assistance recipients provided by municipal welfare offices. The database included information on age, sex, number of family members, household composition, nationality, employment status, and income. Municipal welfare officers collected these data to determine the receipt of public assistance and the amount of monthly minimum income to benefit. Therefore, the database did not contain any missing data. To obtain information on outcomes, we used administrative medical assistance claims data from April 2016 to March 2017. This database included medical, diagnostic procedure combination, dental, pharmacy, and visiting nurses' claims covered by Japan's National Health Insurance Program, which distinguished between inpatient claims and outpatient claims. However, because this administrative medical assistance database was processed and used for the welfare offices' work, resulting in not including raw data of medical claims. Therefore, we could not obtain information on the diagnosis and treatment content in detail.

Each municipality linked the two databases using individual identification codes. The welfare officers of the municipalities agreed to provide anonymized data to the authors via a system company that provided management software for the municipalities' public assistance database. All experiments were performed in accordance with the relevant guidelines and regulations of the Japanese government and the Declaration of Helsinki. The Ethics Committee of Kyoto University Graduate School and the Faculty of Medicine waived the requirement for informed consent. The study protocol was approved by the Ethics Committee of the Kyoto University Graduate School and Faculty of Medicine (approval no. R3356).

### Measurements and variables

#### Outcome variable

From the medical assistance claims data, we identified the first instance of hospitalization among the study participants during the study period. Then we created a binary variable for children with at least one hospitalization during the observation period. Then we calculated cumulative incidence of hospitalization during the observational period. Because information about diagnosis and treatment content was unavailable, we used all‐cause hospitalization as the outcome variable.

#### Explanatory variables

Based on data availability and our previous studies,^20^ we extracted information as of April 2016, including the child's age, sex (male/female), single‐parent family status, guardians' employment status, presence of siblings, timing of receiving public assistance (before birth/ after birth) and nationality (Japanese or other). As a previous study found that the combination of household composition and employment status is associated with healthcare access among public assistance recipients,^23^ we generated a new interaction variable between single parenthood and guardians' employment status, which may influence the time available for children's care (working single parent, nonworking single parent, working nonsingle parent, and non‐working non‐single parent). Age was categorized into four groups according to the national survey of the comprehensive survey of living conditions^24^ as follows: zero, one–four, five–nine, and 10–14 years. Furthermore, we coded municipalities as a dummy variable to adjust for the unmeasured cultural and environmental characteristics of the six municipalities (A–F) and used it as a fixed effect. We also used the variable of whether the children were certified as having mental, intellectual, or physical disabilities. Municipal officials certify disabilities based on the diagnoses of designated physicians and agencies. Households raising children with disabilities received additional benefits from social care.

### Statistical analysis

First, we described the baseline characteristics of the study participants and hospitalized children. Second, we performed univariate modified Poisson regression analysis and calculated the crude cumulative incidence ratio (IR) and 95% confidence interval (CI) of hospitalization for each explanatory variable. The modified Poisson regression using binary outcomes allows for the direct calculation of risk ratios—a widely used approach in epidemiological research due to its significant advantages.^25^ Third, we performed multivariable modified Poisson regression analysis to calculate the multivariable‐adjusted IR of each explanatory variable. Robust error variance was used to calculate the 95% CI. As a robustness check, we conducted a sensitivity analysis, excluding children who received inpatient care at baseline month (Analysis S1). This process allowed us to identify the population at risk of novel hospitalization because we could exclude children receiving long‐term inpatient care from the study participants. There may be some households that have children with severe medical needs, resulting in losing their working income by the children's care and support, then starting to receive public assistance benefits. We could diminish the likelihood of reverse causation by excluding children who received inpatient care in the first observational month. All analyses were performed using STATA MP Version 18 (Stata Corp., College Station, TX, USA).

## RESULTS

We used data from 1990 children from families receiving public assistance. Among them, 998 (50.2%) were girls, 1392 (69.9%) had siblings, and 1917 (96.3%) were Japanese. A total of 498 (25.0%) children were living with a working single parent, 758 (38.1%) children were living with a single parent not working, 402 (20.2%) children were living in a nonsingle‐parent household who were working, and 332 (16.7%) of children were living in a nonsingle‐parent household who were not working. Three (0.2%), 24 (1.2%), and 13 (0.7%) children had mental, intellectual, and physical disabilities, respectively. A total of 532 (26.7%) children were born in public assistance households, and 1458 (73.3%) children started receiving public assistance after their birth. Overall, 91 (4.6%) children were hospitalized during the observation period (Table 1). The incidence proportion of hospitalization across age groups was 16.7% in children aged zero years, 6.7% in those aged 1–4 years, 4.8% in those aged 5–9 years, and 2.6% in those aged 10–14 years. Among children with intellectual and physical disabilities, 12.5% and 30.8% were hospitalized, respectively, while 4.3% of children without disability certificates were hospitalized. The incidence proportion was higher in children living with single parents who are not working and different across municipalities.

**TABLE 1:** Characteristics of study participants and hospitalization rates among children on public assistance.

Character		Total participants (*n* = 1990)	Hospitalization (*n* = 91)
		*n* (%)	*n* (%)
Age	Mean (SD)	8.32 (4.12)	6.07 (4.33)
Age groups	0	66 (3.3)	11, 16.7%
	1–4	356 (17.9)	24, 6.7%
	5–9	686 (34.5)	33, 4.8%
	10–14	882 (44.3)	23, 2.6%
Sex	Boy	992 (49.8)	51, 5.1%
	Girl	998 (50.2)	40, 4.0%
Household statuses	Single parent and working	498 (25.0)	20, 4.0%
	Single parent and not working	758 (38.1)	42, 5.5%
	Nonsingle parent and working	402 (20.2)	18, 4.5%
	Nonsingle parent and not working	332 (16.7)	11, 3.3%
Presence of Siblings	No	598 (30.1)	29, 4.8%
	Yes	1392 (69.9)	62, 4.5%
Receiving Public assistance at birth	No	1458 (73.3)	53, 3.6%
	Yes	532 (26.7)	38, 7.1%
Nationality	Japanese	1917 (96.3)	90, 4.7%
	Other	73 (3.7)	1, 1.4%
Disabilities certificate	None	1950 (98.0)	84, 4.3%
	Mental disability	3 (0.2)	0, 0%
	Intellectual disability	24 (1.2)	3, 12.5%
	Physical disability	13 (0.7)	4, 30.8%
Municipality	A	765 (38.4)	32, 4.2%
	B	627 (31.5)	34, 5.4%
	C	354 (17.8)	11, 3.1%
	D	148 (7.4)	11, 7.4%
	E	77 (3.9)	3, 3.9%
	F	19 (1.0)	0, 0%

In multivariable Poisson regression analyses, the IR of children aged 0, 1–4 years, and 5–9 years was higher than that of children aged 10–14 years (IR 7.46; 95% CI: 3.82–14.57; IR 2.71, 95% CI: 1.56–4.73, IR 1.83, 95% CI: 1.09–3.08, respectively). Children who were certified as having intellectual and physical disabilities had a higher incidence of hospitalizations than children without any disabilities (IR: 4.02, 95% CI: 1.34–12.11; IR: 7.33, 95% CI: 3.04–17.72, respectively) (Table 2). The incidence of hospitalization was slightly higher among children in nonworking single‐parent households (IR: 1.65, 95% CI: 0.88–3.09) and among children in working nonsingle‐parent households (IR: 1.62, 95% CI: 0.78–3.36), whereas the incidence rate was lower among children in working single‐parent households than those two households. The incidence of children's hospitalization varied across municipalities. Sensitivity analysis, excluding the children who were hospitalized at baseline, showed similar results (Table S1).

**TABLE 2:** Hospitalization incidence ratios (IR) by participant characteristics: Crude and multiple Poisson regression results.

Character	Crude IR (95% CI)	Adjusted IR (95% CI)
Age (year, Ref: 10–14)		
0	6.39, (3.26–12.54)	6.49, (2.83–14.84)
1–4	2.59, (1.48–4.52)	2.52, (1.37–4.65)
5–9	1.84, (1.09–3.11)	1.80, (1.06–3.05)
Sex (Ref: Boy)		
Girl	0.83, (0.54–1.27)	0.82, (0.55–1.23)
Household statuses (Ref: Nonsingle parent and not working)		
Single parent and working	1.21, (0.59–2.50)	1.48, (0.73–3.00)
Single parent and not working	1.67, (0.87–3.21)	1.65, (0.88–3.09)
Nonsingle parent and working	1.35, (0.65–2.82)	1.62, (0.78–3.36)
Presence of Siblings under 15 (Ref: No)		
Yes	0.92, (0.60–1.41)	0.90, (0.59–1.36)
Receiving Public assistance at birth (Ref: No)		
Yes	1.96, (1.31–2.95)	1.16, (0.71–1.90)
Nationality (Ref: Japanese)		
Not Japanese	0.29, (0.04–2.07)	0.31, (0.04–2.19)
Disability (Reference: Not certified)		
Mental disability	N/A	N/A
Intellectual disability	2.90, (0.99–8.54)	4.03, (1.32–12.34)
Physical disability	7.14, (3.07–16.58)	7.30, (3.04–17.50)
Municipality (Reference: A)		
B	1.30, (0.81–2.08)	1.38, (0.87–2.19)
C	0.74, (0.38–1.46)	0.86, (0.44–1.67)
D	1.78, (0.92–3.45)	1.89, (0.99–3.61)
E	0.93, (0.29–2.97)	0.93, (0.28–3.11)
F	N/A	N/A

## DISCUSSION

Using data from six municipalities in Japan, we described the characteristics of children receiving public assistance. Of these children, 25.0% were living with a working single parent, 38.1% were living with a single parent not working, 20.2% were living in a nonsingle‐parent household who were working, and 16.7% were living in a nonsingle‐parent household who were not working, and 69.9%of children had siblings. In total, 2.1% of the children were certified as having disabilities. Approximately one quarter of the children were born into households already receiving public assistance, and 4.5% of them experienced hospitalization within a year. The incidence of hospitalization was higher among infants, particularly among children aged 0 years (16.7%). Furthermore, children with certificates of physical or intellectual disabilities experienced a higher incidence of hospitalization compared with children without disability certificates. Among children in single‐parent households, hospitalization rates varied based on the guardian's employment status, with higher rates observed in children whose single parents were not employed. There were regional disparities in hospitalization rates.

Compared to a previous government report,^14^, ^24^ the 1‐year incidence of hospitalization among children receiving public assistance was higher than that in the general population. According to a national survey (Longitudinal Survey of Newborns in the 21st Century), Abe reported that the 1‐year incidence of hospitalization was 12.4% among 0‐year‐old children living above the poverty line.^14^ However, the methodologies used to calculate hospitalization incidence in this study and the national survey differ, and no population‐based data is available that would allow for a direct comparison of hospitalization incidence between recipients and nonrecipients. Therefore, further dataset development and research will be needed to address this gap.

In this study, municipal differences in children's hospitalization rates were identified. Previous studies examining prefectural differences in children's hospitalization in Japan have shown that children living in more rural areas face disadvantages in accessing healthcare and experience higher rates of hospitalization.^26^ Additionally, research highlights the growing disparity in the geographic distribution of pediatricians and accessibility to pediatric care across Japan,^27^, ^28^, ^29^ resulting in regional disparities in child health.^30^ As Sasaki et al. noted, there are highly urbanized areas with a low supply of pediatricians.^27^ In fact, municipalities D and B, which had the highest and second‐highest hospitalization rates in this study, were urbanized areas but had fewer pediatric healthcare institutions per child than other municipalities (Table S1). This lower supply of pediatricians may be contributing to the poorer health outcomes observed in these municipalities. Additionally, regional factors, such as the areal deprivation index or neighborhood socioeconomic status, may contribute to the higher hospitalization rates.^31^ A study conducted in the United States found that pediatric influenza‐associated hospitalization rates were significantly higher in neighborhoods with high poverty levels.^32^ In Japan, areal deprivation is widely recognized as being associated with shorter life expectancy, unhealthy behaviors, and negative health outcomes.^33^

According to previous research,^20^ single parenthood has been identified as a predictor of poor health in children receiving public assistance. In our study, using hospitalization as the outcome and examining the interaction with parental employment status, we found that children living with single parents, as well as those living with working nonsingle parents, were more vulnerable to health risks leading to hospitalization. Although further investigation, including qualitative data, is needed, public health and social welfare sectors focused on maternal and child care should provide targeted health support to these populations. Additionally, medical providers, including pediatricians, need to take a more proactive approach in assessing the risks of hospitalization for these children.

Regarding public assistance policies, the government has launched a health management program for individuals receiving public assistance, mandated by municipal governments.^34^ However, the children who receive public assistance are not the target population for this program. Given the findings of this study, the program should include support aimed at improving children's health issues and provide additional care for children, in alignment with related policies such as “The Child Poverty Act” and policies associated with the Children and Families Agency *(Kodomo‐Katei‐Cho)*. Based on the law, the Japanese government recommends supporting children in need by strengthening community governance within welfare offices, healthcare institutions, nurseries, and schools.^35^ A novel certification for social workers is established (i.e., Child and Family Social Worker: CFSW). Our study indicates that policies should provide more focused support beyond financial aid for children on public assistance to ensure the healthy development of children. However, to design effective support strategies, it is essential to conduct additional analyses using detailed quantitative data, such as disease diagnoses and treatment content, alongside qualitative data on children's and families' living experiences. This would provide a more comprehensive understanding of the barriers and facilitators to care, which cannot be captured by quantitative data alone. The evidence presented in this study, along with the following limitations of existing research and the data revealed here, will likely contribute to the advancement of future research in this area.

Although this study provides important insights using data on children receiving public assistance, which is typically difficult to access and rarely available to the public, we acknowledge several limitations. First, although we used longitudinal data, reverse causation remains a possibility. Some children may develop severe diseases, resulting in financial difficulties and a need for public assistance. However, the results of the analyses, after removing the incidence of hospitalization in the first month of the study period, indicated that reverse causation did not alter our conclusions. Second, we could not establish a causal relationship between receiving public assistance and children's health, because children's data before receiving public assistance and a valid comparable dataset among nonusers of public assistance are not well‐established. Third, we could not obtain data on important factors that were not evaluated in this study, including the diagnosis, severity of disease, and treatment contents, which may have biased our findings. Future studies should use the raw claims data regarding the medical use among the recipients. Fourth, the generalizability of our findings is limited because the municipalities included in this study may not represent all municipalities in Japan. Finally, we were unable to determine whether the relationships observed in this study were unique to children receiving public assistance. To effectively address the adverse impact of child poverty, it is essential to establish evidence on impoverished households not covered by public assistance. However, conducting social surveys on these populations presents significant challenges. Potential solutions include utilizing data on children supported by the School Expense Subsidy (*Shugaku‐Enjo*), the Free and Low‐Cost Medical Care program (*Muryo‐Teigaku‐Shinryo*), or those exempt from residential taxes, which can be identified through municipal data or medical records. A survey of these children, along with the development of a dataset that links resident registry data and medical data, is necessary to further advance this research. Despite the abovementioned limitations, this is the first study to identify the incidence of hospitalization and its associated factors among children receiving public assistance in Japan, using the existing municipal public assistance data, which are somewhat difficult to reach using a standard social survey.

In conclusion, children receiving public assistance in Japan may face higher risks of hospitalization. Differences in hospitalization rates were also observed across children's household statuses and residential areas. These findings highlight the need for targeted healthcare support to address the specific needs of these children; however, additional research using more detailed quantitative and qualitative data is essential. Future studies should also explore the relationship between child poverty and health outcomes by including data from both children receiving public assistance and those not covered by such programs.

## AUTHOR CONTRIBUTIONS

DN, SK, KU, and NK conceptualized and designed the study. DN analyzed the data and prepared the manuscript. SK, KU, and NK reviewed the manuscript. DN finalized the manuscript. All authors have read and approved the final version of the manuscript.

## CONFLICT OF INTEREST STATEMENT

Daisuke Nishioka, Shiho Kino, and Keiko Ueno declare no competing interests associated with this manuscript. Naoki Kondo collaborated with Kitanihon Computer Service Co., Ltd. (KITACOM), from which the data used in this study were obtained. Naoki Kondo received research funds and scholarship donations from KITACOM. The research funding bodies had no discretion or involvement in the study protocol, analysis, interpretation of the results, or submission of this manuscript.

## Supporting information


Data S1.

## Data Availability

The data used in this study were obtained from participating municipalities in Japan. However, there are restrictions regarding the availability of these data, which were used under the license for the current study and are not publicly available. The data are available from the authors upon reasonable request, with permission from the municipalities.
