# Supplementary material for: Characteristics and hospitalizations among children on public assistance in Japan: A population‐based cohort study
Source: Pediatr Int. 2025 Jun 10;67(1):e70005. doi: 10.1111/ped.70005 (PMC12150824; doi:10.1111/ped.70005)
Supplement: Supplementary file 1 — Data S1. [file PED-67-e70005-s001.docx]

Table S1. Summary of Pediatric Healthcare and Welfare Statistics in the Municipalities Where Study Participants Reside

| Municipality | Area (km²) | Population (2015) | Population Under 15 (2015) | Public Assistance Rate (%) | Number of Single-Parent Households (2015) | Pediatric Healthcare Institutions (2015) | Pediatric Healthcare Institutions per a thousand Children Under 15 (2015) | Child Medical Expense Subsidy |
| --- | --- | --- | --- | --- | --- | --- | --- | --- |
| A | 70 | 190,000 | 29,000 | 3.2 | 1,700 | 29 | 1 | Reduction at counter (¥500), pre-application required, with limits on amount and frequency |
| B | 80 | 190,000 | 31,000 | 2.3 | 1,500 | 21 | 0.7 | Reduction at counter (¥500), pre-application required, with limits on amount and frequency |
| C | 20 | 180,000 | 24,000 | 2.8 | 1,800 | 27 | 1.1 | Reduction at counter (¥200), pre-application required |
| D | 60 | 100,000 | 14,000 | 1.8 | 800 | 10 | 0.7 | Reduction at counter (¥500), pre-application required, with limits on amount and frequency |
| E | 190 | 80,000 | 9,000 | 2.2 | 400 | 13 | 1.4 | Reimbursement system, application required |
| F | 790 | 50,000 | 5,000 | 1.4 | 200 | 3 | 0.6 | Reimbursement system, application required |

*Note: Municipality names have been anonymized, and values have been rounded to obscure exact figures of municipalities.

Supplementary Table S2. Incidence ratios (IR) and 95% confidence intervals (CI) for post -baseline hospitalization- multiple Poisson regression results

| Character |  | Adjusted IR (95%CI) |
| --- | --- | --- |
| Age (Ref: 10-14) |  |  |
| 0 |  | 6.02, (2.45-14.76) |
| 1-4 |  | 2.33, (1.21-4.49) |
| 5-9 |  | 1.58, (0.89-2.81) |
| Sex (Ref: Boy) |  |  |
| Girl |  | 0.73, (0.46-1.15) |
| Household statuses (Ref: Non-single-parent and not working) |  |  |
| Single-parent and working |  | 1.30, (0.63-2.68) |
| Single-parent and not working |  | 1.27, (0.67-2.43) |
| Non-single-parent and working |  | 1.54, (0.74-3.20) |
| Presence of Siblings under 15 (Ref: No) |  |  |
| Yes |  | 0.77, (0.49-1.21) |
| Receiving Public assistance at birth (Ref: No) |  |  |
| Yes |  | 1.24, (0.73-2.11) |
| Nationality (Ref: Japanese) |  |  |
| Not Japanese |  | 0.37, (0.05-2.62) |
| Disability (Reference: Not certified) |  |  |
| Mental disability |  | N/A |
| Intellectual disability |  | 3.11, (0.81-11.95) |
| Physical disability |  | 8.70, (3.59-21.08) |
| Municipality (Reference: A) |  |  |
| B |  | 1.50, (0.90-2.48) |
| C |  | 0.98, (0.48-2.00) |
| D |  | 2.29, (1.14-4.62) |
| E |  | 0.80, (0.19-3.48) |
| F |  | N/A |
